# Recurrence after Successful Treatment of Multidrug-Resistant Tuberculosis in Taiwan

**DOI:** 10.1371/journal.pone.0170980

**Published:** 2017-01-26

**Authors:** Meng-Yu Chen, Yi-Chun Lo, Wan-Chin Chen, Kwei-Feng Wang, Pei-Chun Chan

**Affiliations:** 1 Office of Preventive Medicine, Centers for Disease Control, Taipei, Taiwan; 2 Division of Chronic Infectious Diseases, Centers for Disease Control, Taipei, Taiwan; 3 Institute of Epidemiology and Preventive Medicine, College of Public Health, National Taiwan University, Taipei, Taiwan; 4 Division of Pediatric Infectious Diseases, Department of Pediatrics, National Taiwan University Hospital, College of Medicine, National Taiwan University, Taipei, Taiwan; Agencia de Salut Publica de Barcelona, SPAIN

## Abstract

Recurrence after successful treatment for multidrug-resistant tuberculosis (MDR-TB) is challenging because of limited retreatment options. This study aimed to determine rates and predictors of MDR-TB recurrence after successful treatment in Taiwan. Recurrence rates were analyzed by time from treatment completion in 295 M DR-TB patients in a national cohort. Factors associated with MDR-TB recurrence were examined using a multivariate Cox regression analysis. Ten (3%) patients experienced MDR-TB recurrence during a median follow-up of 4.8 years. The overall recurrence rate was 0.6 cases per 1000 person-months. Cavitation on chest radiography was an independent predictor of recurrence (adjusted hazard ratio [aHR] = 6.3; 95% CI, 1.2–34). When the analysis was restricted to 215 patients (73%) tested for second-line drug susceptibility, cavitation (aHR = 10.2; 95% CI, 1.2–89) and resistance patterns of extensively drug-resistant TB (XDR-TB) or pre-XDR-TB (aHR = 7.3; 95% CI, 1.2–44) were associated with increased risk of MDR-TB recurrence. In Taiwan, MDR-TB patients with cavitary lesions and resistance patterns of XDR-TB or pre-XDR-TB are at the highest risk of recurrence. These have important implications for MDR-TB programs aiming to optimize post-treatment follow-up and early detection of recurrent MDR-TB.

## Introduction

Multidrug-resistant tuberculosis (MDR-TB), defined as tuberculosis caused by a strain of *Mycobacterium tuberculosis* resistant to at least isoniazid and rifampin, remains a global concern. Over the past several years, a growing number of studies have shown that recurrence after successful treatment of MDR-TB was not uncommon [1–15] (S1 Appendix). Reported recurrence rates vary worldwide from 0% after 2 years of follow-up [5,13,15] to 8.5% after 8 years of follow-up [6]. Managing patients with MDR-TB recurrence represents a challenge for medical and public health professionals because of limited retreatment options and continuing threat to close contacts.

In Taiwan, a national program for MDR-TB organized according to the World Health Organization (WHO) guidelines [16,17], named Taiwan MDR-TB Consortium (TMTC), was launched in May 2007 [18]. It was implemented under the Taiwan National Tuberculosis Program and augmented the conventional model of directly observed treatment, short course-plus (DOTS-Plus) for MDR-TB patients. Between 2007 and 2011, the number of incident MDR-TB patients remained unchanged, accounting for 1% of TB patients and 6% of retreated patients [18,19]. The rate of treatment success increased from 61% to 82% after the implementation of the TMTC [20]. The number of prevalent MDR-TB patients also decreased from 440 in May 2007 to 210 in April 2014 [19]. However, whether long-term outcomes for patients enrolled in the TMTC are also favorable is unknown. Moreover, studies on the predictors of MDR-TB recurrence are few and limited by lack of adjustment for potential confounders [1,4,6–9]. Elements necessary for identifying recurrences such as at-risk populations and both the frequency and duration of follow-up have not been determined. We aimed to use the national surveillance data to estimate MDR-TB recurrence rates under the TMTC and to evaluate predictors of MDR-TB recurrence in Taiwan.

## Methods

### Study setting and procedures

Since 2007 the TMTC has provided medical care and operated DOTS-Plus project to MDR-TB patients through five professional therapeutic teams around the country. Designated observers and nurses employed by therapeutic teams delivered individualized second-line anti-TB drugs to patients, with enablers and incentives to support facing lengthy treatment of MDR-TB. The DOTS-Plus team staff reported adverse events or other medical conditions to the physician in charge of patients to concur the frequent encountered side effects of treatment [18,20]. Pulmonary MDR-TB patients with positive culture results who had not completed treatment by January 2007 were informed of the TMTC and given the option to participate. The enrolment rate of MDR-TB patients in the TMTC was 90% while refusal and relocation abroad were the two main reasons why patients did not receive care of the TMTC [18]. Baseline demographics, concomitant diseases such as diabetics and cancer, disease severity, drug resistance, treatment course and outcomes of patients participating in the TMTC were recorded in the TMTC registry. Although human immunodeficiency virus (HIV) prevalence in Taiwan is low (0.16% in 2011) [21] and HIV testing was not mandatory for MDR-TB patients, the TMTC was encouraged to perform testing for all enrolled patients since 2009.

Drug susceptibility testing (DST) was first done on culture-positive isolates at laboratories of designated hospitals. Culture-positive isolates were then submitted to the Reference Laboratory of Mycobacteriology at Taiwan Centers for Disease Control (TCDC) for confirmation of MDR-TB and DST to second-line drugs. The TCDC Reference Laboratory of Mycobacteriology used the Middle-brook 7H11 agar proportion method to perform DST for all drugs, including fluoroquinolones (ofloxicin, ciprofloxacin, levofloxacin, moxifloxicin) and second-line injectable drugs (kanamycin, amikacin, capreomycin) [22]. Early in the program not all isolates were tested for second-line DST. In the latter stages, such testing was done in almost all collected isolates [20]. Regimens were routinely adjusted according to results of in vitro susceptibility testing as WHO guidelines [16]. Since individualized instead of standardized regimen was applied, the treatment duration could be tailored by clinical response, mainly decided by physicians in charge of the patient.

Treatment outcomes were documented based on WHO standard definitions [17]: *cured* meant patients completed treatment according to program protocol and had at least five consecutive negative cultures from samples collected at least 30 days apart in the final 12 months of treatment; *completed* meant patients completed treatment according to program protocol but did not meet the definition for cured because of lack of bacteriological results (i.e. fewer than five cultures performed in the final 12 months of treatment). *Treatment success* included both cured and treatment completed. As all the other pulmonary TB patients, physicians were recommended to provide post-treatment follow-up for MDR-TB patients (i.e. chest radiograph and sputum collection every 6 months for a year after completion of treatment and then yearly thereafter) [23]. No other routine monitoring after completion of treatment was recommended specifically for MDR-TB patients.

### Study design and data collection

We performed a cohort study among all laboratory-confirmed MDR-TB patients who were registered at the TMTC from May 2007 to December 2010 and achieved treatment success. The primary outcome of this study was the occurrence of active MDR-TB during follow-up after treatment success, defined as MDR-TB recurrence. Episodes of recurrent MDR-TB were identified when patients had at least one positive sputum culture for MDR-TB (with DST confirmation) collected at least 3 months after the end of successful treatment. Patients with positive sputum culture within 3 months after the end of treatment were considered *treatment incomplete* and not classified as recurrent MDR-TB. *Time to recurrence* was defined as the time between the documented end date of the treatment for MDR-TB and the date of positive sputum samples collection for recurrent MDR-TB. The cohort was followed up from the end of treatment until disease recurrence, death, and emigration or until 20 September 2014, whichever came first. We used unique national identification number and linked to routinely collected National Tuberculosis Registry data to identify recurrent episodes. All other data were collected from the TMTC registry database.

### Statistical analysis

We calculated the percentage of patients who had a recurrence of MDR-TB and the timing of recurrence. We evaluated predictor variables associated with time to recurrence. Categorical variables are reported as proportions, and the comparisons between groups were carried out by using the chi-square test or Fisher’s exact test. All statistical tests were 2-sided, no multiple comparison adjustment was made, and p<0.05 was considered statistically significant. We used Kaplan-Meier estimates to construct a cumulative recurrence-free curve and Cox proportional hazard models to conduct univariate and multivariate analyses. We counted the duration of follow-up from the end of treatment to recurrence or to the end of observation. We developed a full model including all potential explanatory variables and used a forced entry method, in which all variables were entered in a single step to identify factors independently associated with recurrence.

Results of DST to second-line drugs (fluoroquinolones and second-line injectable drugs) were missing for 27% of cases; hence, this variable was not included in the full model of multivariate analysis. However, as drug resistance patterns have been previously linked to treatment outcomes of MDR-TB [24], we enrolled patients who had undergone second-line DSTs in a subgroup analysis to assess its impact on recurrence.

All the analyses were performed using the SPSS statistical package, version 20.0 (SPSS Inc. Chicago, IL, USA) and proportional hazards assumption test was completed by using the SAS, version 9.3 (SAS Institute Inc., Cary, NC, USA).

### Ethics statement

The study was approved by TCDC. As it was deemed public health surveillance, it was exempted from ethics approval by the Institutional Review Board of TCDC.

## Results

**Fig 1:**
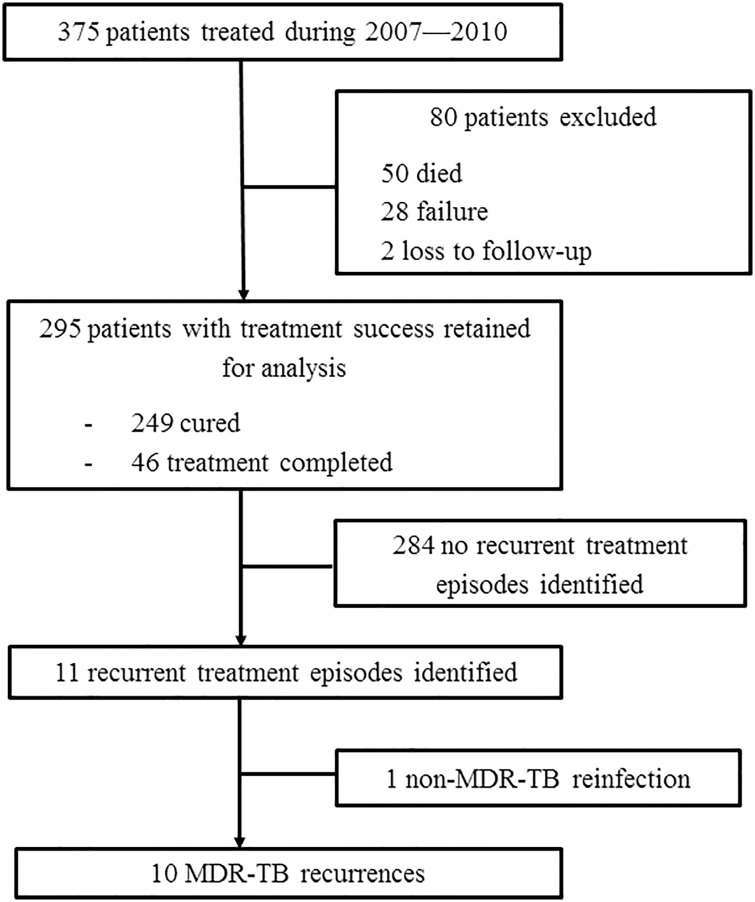
Flow chart for number of patients included in and excluded from the study. Treatment success, with outcomes as cure or treatment completion; Cured, completing treatment according to program protocol and having at least five consecutive negative cultures from samples collected at least 30 days apart in the final 12 months of treatment; Treatment completed, completing treatment according to program protocol but not meeting the definition for cure because of lack of bacteriological results (i.e. fewer than five cultures performed in the final 12 months of treatment). Failure, two or more of the five cultures recorded in the final 12 months of therapy are positive, or if any one of the final three cultures is positive. Loss to follow-up, failure to obtain contact with the patient before the end of treatment so that treatment outcome is not known. Recurrence, least one positive sputum culture for MDR-TB collected at least 3 months after the end of successful treatment. MDR-TB, multidrug-resistant tuberculosis; TMTC, Taiwan MDR-TB Consortium.

There were 375 MDR-TB patients registered at the TMTC between May 2007 and December 2010, of whom 295 achieved treatment success (249 were cured, and 46 completed treatment) (Fig 1). These 295 patients with successful outcomes comprised our cohort of analysis. The characteristics of the 295 patients were summarized in Table 1. Among these 295 patients, 228 (77%) were male. The median age at the initiation of MDR-TB treatment was 48 years (interquartile range [IQR], 37–59 years). Of the 191 patients who underwent HIV testing, four (2.1%) were HIV positive. Patient classification in the registry showed that 62% had history of previous anti-TB treatment (relapse, treated after loss to follow-up, or treated after failure). Patients with previous treatment history were more likely to have cavitation on the chest radiograph compared to newly diagnosed patients (p = 0.005).

**Table 1:** Baseline characteristics of multidrug-resistant tuberculosis patients with treatment success (n = 295).

	Patients n	MDR-TB recurrence n (%)	Univariate HR (95% CI)	P value
Total	295	10 (3)		
Gender				
Male	228	9 (4)	2.8 (0.3–22)	0.33
Female	67	1 (1)	Reference	
Age groups (years)				
>60	66	2 (3)	0.6 (0.1–3.1)	0.52
35–60	163	4 (3)	0.4 (0.1–1.7)	0.23
<35	66	4 (6)	Reference	
Aboriginal				
Yes	61	0 (0)	—	—
No	234	10 (4)		
BMI (kg/m^2^)				
<18.5	56	1 (2)	0.9 (0.1–13)	0.91
18.5–25	195	8 (4)	1.9 (0.2–15)	0.56
≥25	44	1 (2)	Reference	
Diabetes mellitus				
Yes	92	5 (5)	2.2 (0.6–7.6)	0.21
No	203	5 (3)	Reference	
Hypertension				
Yes	51	1 (2)	0.6 (0.1–4.2)	0.55
No	244	9 (4)	Reference	
Hepatitis B				
Yes	23	0 (0)	—	—
No	272	10 (4)		
Hepatitis C				
Yes	25	1 (4)	1.3 (0.2–10)	0.82
No	270	9 (3)	Reference	
HIV infected (n = 191)				
Yes	4	0 (0)	—	—
No	187	7 (4)		
Patient classification				
Treatment after failure of previous treatment	75	2 (3)	1.5 (0.2–10)	0.70
Treatment after loss to follow-up	17	1 (6)	3.5 (0.3–39)	0.30
Relapse	92	5 (6)	3.1 (0.6–16)	0.18
New	111	2 (2)	Reference	
Cavitation on initial CXR				
Yes	124	8 (7)	5.5 (1.2–26)	0.03
No	171	2 (1)	Reference	
Sputum smear positivity at time of diagnosis				
Yes	174	6 (3)	1.0 (0.3–3.7)	0.96
No	121	4 (3)	Reference	
Second-line DST (n = 215)				
XDR	15	1 (7)	2.6 (0.3–23)	0.39
Pre-XDR	43	3 (7)	2.7 (0.6–12)	0.19
MDR only	157	4 (3)	Reference	
Treatment delay ^a^				
Yes	77	4 (5)	1.9 (0.5–6.6)	0.34
No	218	6 (3)	Reference	
Culture conversion before initiating SLD				
Yes	73	3 (4)	1.3 (0.3–4.9)	0.73
No	222	7 (3)	Reference	
Time from initiating SLD to culture conversion				
≥2 months	117	3 (3)	0.6 (0.2–2.5)	0.52
<2 months	178	7 (4)	Reference	
Treatment outcome				
Completed	46	3 (7)	2.4 (0.6–9.2)	0.21
Cured	249	7 (3)	Reference	

BMI, body mass index; CI, confidence interval; CXR, chest radiography; DST, drug susceptibility test; HR, hazard ratio; MDR-TB, multidrug-resistant tuberculosis (resistance to at least isoniazid and rifampin); MDR only, MDR-TB but susceptible fluoroquinolones and second-line injectable drugs; pre-XDR, MDR-TB plus resistance to any fluoroquinolone or any second-line injectable drug; SLD, second-line drugs (include fluoroquinolones and second-line injectable drugs); XDR, extensively drug-resistant tuberculosis (MDR-TB plus resistance to any fluoroquinolone and any second-line injectable drug).

^a^ The lag between sputum collection of MDR-TB and start of second-line drugs >120 days.

A total of 124 patients had resistance.to streptomycin, representing 43% of those tested. Two hundred and fifteen patients had results of second-line DST available. Of these, 15 patients (7%) had strains of extensively drug-resistant tuberculosis (XDR-TB; MDR plus resistance to fluoroquinolone and second-line injectable drugs); 43 (20%) had strains of pre-extensively drug-resistant tuberculosis (pre-XDR-TB; MDR plus resistance to fluoroquinolone or second-line injectable drugs) (Table 1).

Among the 295 patients included, all received regimens with a fluoroquinolone and 283 (96%) received an injectable drug during the entire course of treatment. Treatment duration over 24 months was documented in 108 (37%) patients, which was more common in patients without inclusion of an injectable drug in the initial MDR-TB regimens (34/63) than among patients with injectable drugs included at treatment initiation (74/232) (p = 0.001).

### Recurrence of MDR-TB

**Fig 2:**
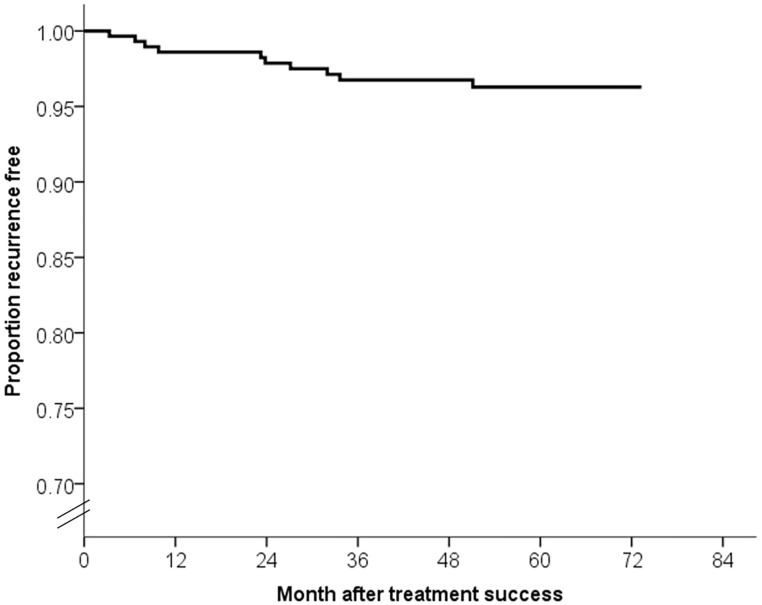
Recurrence free survival curve for multidrug-resistant tuberculosis patients (n = 295).

The median duration of post-treatment follow-up was 4.8 years (IQR, 4.1–5.3 years). By the end of the observation period, 10 (3.4%) patients had MDR-TB recurrence. Four of 10 recurrences occurred in the first year, two in the second year, and the other four occurred more than 2 years after treatment success (Fig 2). The incidence rate of recurrence was 1.2 cases per 1000 person-months (95% confidence interval [CI], 0.5–3.0 cases per 1000 person-months) in the first 12 months after treatment success and 0.6 cases per 1000 person-months (95% CI, 0.2–2.2 cases per person-months) in months 13–24 after treatment success. The overall incidence rate of recurrence was 0.6 cases per 1000 person-months (95% CI, 0.4–1.2 cases per 1000 person-months). The demographics, drug resistance patterns and treatment courses of the 10 patients experiencing recurrence were summarized in Table 2. Results of second-line DST in original and recurrent strains were both known for 6 patients (3 MDR-TB, 2 pre-XDR-TB and 1 XDR-TB) (Table 2 and S2 Table). None of them acquired additional resistance to fluoroquinolones or second-line injectable drugs between two episodes of MDR-TB. By the end of observation, six patients had achieved treatment success for recurrent episodes, two were still under treatment, one patient had died, and one had failed. The patient who failed treatment is currently categorized as a chronic case, attributed to poor response due to interactions between anti-psychotic medications and second-line anti-TB drugs.

**Table 2:** Characteristics and treatment outcomes of 10 patients with multidrug-resistant tuberculosis recurrence.

		Initial episode									Recurrent episode			
Case No.	Gender	Age (y)	Status at time of initiating treatment (patient classification)	Sputum smear at time of diagnosis	Cavitation on CXR	Second-line DST^a^	Time to conversion (m)^b^	Treatment outcome	Treatment duration (m)	Interval to recur (m)	Sputum smear at time of diagnosis	Cavitation on CXR	Second-line DST^a^	Treatment outcome
1	M	18	New	Negative	Yes	NA	1.8	Cure	21.3	8.0	Negative	No	Pre-XDR	Complete
2	M	61	New	Negative	No	Pre-XDR	1.3	Cure	33.8	9.8	Negative	No	NA	Death
3	M	24	Relapse	Positive	Yes	MDR only	19.6	Complete	29.4	32.0	Positive	Yes	MDR only	Cure
4	M	35	Relapse	Negative	No	MDR only	0.3	Complete	20.3	23.8	Negative	No	MDR only	Cure
5	M	41	Relapse	Positive	Yes	Pre-XDR	^c^	Cure	41.9	3.3	Positive	No	Pre-XDR	Chronic
6	F	49	Relapse	Positive	Yes	XDR	19.3	Cure	40.6	6.7	Positive	Yes	XDR	Cure
7	M	61	Relapse	Positive	Yes	MDR only	1.3	Cure	13.8	33.6	Positive	No	NA	Cure
8	M	46	Return from loss to follow-up	Positive	Yes	MDR only	1.7	Complete	25.1	27.1	Positive	Yes	MDR only	Under treatment
9	M	47	After failure	Positive	Yes	MDR only	0.3	Cure	29.2	23.2	Positive	Yes	NA	Cure
10	M	53	After failure	Positive	Yes	Pre-XDR	4.2	Cure	35.4	51.1	Positive	No	Pre-XDR	Under treatment

CXR, chest radiography; DST: drug susceptibility test; NA, not available; MDR-TB, multidrug-resistant tuberculosis (resistance to at least isoniazid and rifampin); MDR only, MDR-TB, but susceptible to fluoroquinolones and second-line injectable drugs; pre-XDR, MDR-TB plus resistance to any fluoroquinolone or any second-line injectable drug; XDR, extensively drug-resistant tuberculosis (MDR-TB plus resistance to any fluoroquinolone and any second-line injectable drug).

^a^ See the full susceptibility results on S2 Table.

^b^ Time from initiating second-line drugs (fluoroquinolones and second-line injectable drugs) to culture conversion.

^c^ Case 5 had conversion before receiving second-line drugs.

In univariate analysis, patients who had cavitation on chest radiography at the time of diagnosis had a 5-fold increase in the risk of MDR-TB recurrence (hazard ratio [HR], 5.5 [95% CI, 1.2–26]; p = 0.03; Table 1). Treatment duration more than 24 months was not associated with recurrence (HR, 3.6 [95% CI, 0.9–14], p = 0.07). In multivariate analysis with covariates adjusted for disease severity and treatment outcomes (proportional hazards assumption test, p = 0.597), cavitation on chest radiography remained independently associated with an increased risk of MDR-TB recurrence (adjusted hazard ratio [aHR], 6.3 [95% CI, 1.2–34]; p = 0.03; S1 Table).

### Subgroup analysis

In our cohort, 215 patients (73%) had results for second-line DST and 80 (27%) did not. Availability of results of second-line DST was associated with sputum smear positivity (p = 0.03) and inversely proportional to culture conversion before the use of second-line drugs (p<0.001) (Table 3). Otherwise, no significant difference was observed between patients with and without results of second-line DST. Among the 215 patients with second-line DST results, 8 patients had MDR-TB recurrence. In multivariate analysis (proportional hazards assumption test, p = 0.602), drug resistance patterns of XDR-TB or pre-XDR-TB were independently associated with an increased risk of recurrence (HR, 7.3 [95% CI, 1.2–44]; p = 0.03). Cavitation on chest radiography was still significantly associated with an increased risk of recurrence (HR, 10.2 [95% CI, 1.2–89]; p = 0.04) (S1 Table).

**Table 3:** Characteristics of multidrug-resistant tuberculosis patients with treatment success, by availability of results of second-line drug susceptibility test.

	Second-line DST results		p value
	Available (n = 215)	Unavailable (n = 80)	
Gender—male	161 (75)	67 (84)	0.11
Age groups (years)			
>60	49 (23)	17 (21)	0.75
35–60	116 (50)	47 (59)	
<35	50 (23)	16 (20)	
Aboriginal	44 (21)	17 (21)	0.88
BMI (kg/m^2^)			
<18.5	36 (17)	20 (25)	0.21
18.5–25	148 (69)	47 (59)	
≥25	31 (14)	13 (16)	
Diabetes mellitus	67 (31)	25 (31)	0.99
Hypertension	38 (18)	13 (16)	0.77
Hepatitis B	20 (9.3)	3 (3.8)	0.11
Hepatitis C	22 (10)	3 (3.8)	0.08
HIV infected (n = 191)	3/151 (2.0)	1/40 (2.5)	1.00^a^
Patient classification			0.48
Treatment after failure of previous treatment	58 (27)	17 (21)	
Treatment after loss to follow-up	10 (4.7)	7 (8.8)	
Relapse	67 (31)	25 (31)	
New	80 (37)	31 (39)	
Cavitation on initial CXR	95 (44)	29 (36)	0.22
Sputum smear positivity at time of diagnosis	135 (63)	39 (49)	0.03
Treatment delay ^b^	59 (27)	18 (23)	0.39
Culture conversion before initiating SLD	41 (19)	32 (40)	<0.001
Time from initiating SLD to culture conversion ≥2 months	87 (41)	30 (38)	0.64
Treatment outcome of initial episode as completed	31 (14)	15 (19)	0.36

Data are presented as n (%).

BMI, body mass index; CXR, chest radiography, DST, drug susceptibility test; SLD, second-line drugs (include fluoroquinolones and second-line injectable drugs).

^a^ Comparison using Fisher’s exact test.

^b^ The lag between sputum collection of MDR-TB and start of second-line drug >120 days.

## Discussion

In this nationwide study with a median duration of follow-up of 4.8 years, 10 (3.4%) of the 295 MDR-TB patients experienced MDR-TB recurrence. Incidences of recurrence in the first 12 months and between months 13–24 of follow-up were lower than or comparable to those reported from the MDR-TB program in Peru [1,2] and Russia [7]. Such low rates of recurrence—either in terms of recurrence proportion or incidence rate—demonstrate the effectiveness of our MDR-TB program, characterized by government-organized, hospital-initiated, and patient-centered care [20].

The low recurrence rate in our study can be explained by at least two reasons. First, MDR-TB patients in our cohort were managed by a consortium of specialized treatment facilities where better adherence and more aggressive follow-up after treatment were more likely to achieve compared to care under traditional community service. The effective drug administration may therefore lead to a more favorable treatment outcome and lower risk for recurrence. Second, the variation of case definitions and follow-up periods in different MDR-TB programs may affect the estimates of recurrence rates. It should be noted that in this study we included only patients with full culture confirmation of MDR-TB as recurrence while in previous studies case definition may include some patients who had re-initiation of treatment but no culture confirmation or DST available for the isolates from recurrent episodes [1,3,7]. Our strict case definition may not be the most sensitive definition but more accurately reflect the true burden of recurrence diseases. Moreover, we found DST results for fluoroquinolones and second-line injectable drugs did not differ between the cultures from two episodes, implying that the observed recurrence rate in this study can be regarded as an estimate of the relapse rate, an important measure of treatment efficacy. Although it would be preferable to use molecular genotyping to differentiate true relapse from reinfection [7,25] such analyses are rarely performed extensively under programmatic conditions [26].

The recommendation of frequency and duration for follow-up of MDR-TB patients has not been well established. Most MDR-TB programs conducted regular follow-up within 2 years after treatment completion for MDR-TB [3,10,13–15]. The study in Peru suggested follow-up for a second year might not be warranted [2] while the teams from Estonia and Russia recommended prolonged follow-up be needed for early detection and treatment of recurrence [6,7]. The recurrence rate in our study peaked during the first year of follow-up, supporting Gelmanova and colleagues’ assumption that MDR-TB relapse typically occurs soon after completion of treatment [7]. Moreover, a number of recurrences in our study occurred beyond the second year of follow-up. This implies the prolonged risk of recurrence in MDR-TB patients even when treatment success has been achieved. We therefore recommend post-treatment follow-up for more than 2 years for MDR-TB patients as TBNet consensus [27]. Patients should also be informed of the long-term risk and of the signs and symptoms of recurrence, especially if MDR-TB programs cannot provide long-term, active follow-up.

It would be more feasible to have predictors for MDR-TB recurrence to identify susceptible patients and minimize the resource burden of post-treatment follow-up. We found that cavitation on chest radiography at the start of treatment was important in predicting MDR-TB recurrence. The occurrence of cavitary lesions in MDR-TB patients ranged widely in previous studies (from 24% to 69%) [1,4,6,12–14]. In our cohort 42% had chest cavitation, possibly attributable to the fact that 62% of our patients had previously received treatment. Chest radiographic findings are linked to severity of TB, and the association between cavitary disease and delay in diagnosis is well documented [28]. The presence of cavities has also been considered as an important risk factor for recurrent tuberculosis [29]. Probable explanations for this association include poor penetration by anti-TB drugs into the cavity [29,30], high bacilli load or suboptimum dosing in patients with cavitation leading to reduced drug effectiveness [31], and increased propensity of mycobacteria to infect previously damaged lung tissue [29,32]. Genetic components to the host immune response to MDR-TB may also predispose some patients to disease recurrence [31]. We therefore propose MDR-TB patients with cavitation be prioritized for post-treatment follow-up to detect recurrence timely. Alternatively, advanced TB diseases like cavitation can be prevented through early case-finding, early diagnosis and a prompt start to effective treatment [17,31]. Whether prolonged treatment courses or aggressive regimens [1] provide benefit in reducing recurrence in patients with cavitation requires further evaluation.

We also found that pre-XDR-TB or XDR-TB patients were more likely to experience disease recurrence, suggesting that treatment regimens for patients with higher levels of drug resistance were inadequate. The findings are consistent with those of our earlier study [20], showing that resistance to quinolones or XDR-TB was associated with lower probability of treatment success. Pre-XDR-TB and XDR-TB are known to be risk factors for poor treatment outcomes [33–35]. Many clinical and programmatic factors may contribute to suboptimal treatment in patients with extensive drug resistance: limited effective drug options, shortened treatment duration due to adverse effects of toxic second-line antibiotics, delays in second-line DST, and lack of supply of later-generation or new anti-TB drugs [1,36]. With development of new MDR-TB medications such as Bedaquiline and Delamanid [37], lower toxicity and higher adherence to treatment are anticipated compared to pre-existing second-line medications. Interventions to minimize exposure to ineffective treatment, careful management of adverse events during treatment, and rapid DST are crucial to reduce recurrence in these difficult-to-treat groups.

Our study has several limitations. First, although MDR-TB recurrence in our study was confirmed by culture and DST, molecular genotyping was not performed in all the recurrent paired strains, which may have resulted in overestimation of recurrence through inadvertent inclusion of reinfection with new MDR-TB strains [25,38]. However, there is little chance of reinfection because the prevalence of MDR-TB was low and declining during the follow-up period of this cohort. Moreover, from our ongoing research of molecular genotyping (data not reported) we found that the strains of two episodes of MDR-TB were identical in eight patients (the strains in the other two patients were not available). Therefore the recurrences in our patients were more likely to attribute to relapse and the recurrence reported in this study was close to the true relapse rate. Second, it is likely that not all patients with recurrence were identified because clinical follow-up after treatment completion is not mandatory in our program. Therefore, the actual number of patients with recurrence may be underestimated. However, we linked data from the National TB registry to identify any new notification after the end of treatment, where notifications have been reinforced by the government policy of ‘no-notification-no-reimbursement’ since 1997 [39]. Thus, the probability of patients with recurrence not identified by our surveillance systems should be low. Third, the relatively small number of recurrences, however, reduced the power of the analysis to detect predictors or significant interactions. Finally, because MDR-TB patients in this cohort were older (with median age 48 years) and less co-infected with HIV (2%), our findings may not be applicable to other MDR-TB programs in which patients are young and facing high HIV burden.

In conclusion, our study showed that most MDR-TB patients remained recurrence-free for 5 years after treatment success under the TMTC. Our analysis found specific predictors of recurrence including cavitation on the initial chest radiograph and resistance patterns of XDR-TB or pre-XDR-TB. Active follow-up of MDR-TB patients for more than 2 years after treatment completion is a reasonable way to detect late recurrence, but the optimal frequency and content of follow-up require further investigation.

## Supporting Information

S1 Appendix Methods and results of literature review of multidrug-resistant tuberculosis recurrence. (DOCX)

S1 Table Multivariate analyses of potential predictors of multidrug-resistant tuberculosis recurrence (n = 295). (DOCX)

S2 Table Detailed information of drug susceptibility results in 10 patients with multidrug-resistant tuberculosis recurrence. (DOCX)
