# Supplementary material for: Recurrence after Successful Treatment of Multidrug-Resistant Tuberculosis in Taiwan
Source: PLoS One. 2017 Jan 26;12(1):e0170980. doi: 10.1371/journal.pone.0170980 (PMC5270331; doi:10.1371/journal.pone.0170980)
Supplement: S1 Appendix — (DOCX) [file pone.0170980.s001.docx]

**Text S1. Methods and results of literature review of multidrug-resistant tuberculosis recurrence**

We conducted a review in which data were identified by using PubMed. The search terms ‘Tuberculosis’, ‘Multidrug-resistant’ and ‘recurrence’ (in MeSH or in title/abstract) were used to search for cohort studies. Neither language restriction nor publication dates were imposed on our searches. The bibliographies of key studies were also reviewed to identify other relevant articles. The following inclusion criteria were used: reporting recurrence (or relapse) after successful treatment, and duration of follow-up ≥24 months. Two investigators extracted information from the eligible studies on outcome (recurrence) definition, recurrence rate (or proportion), follow-up duration and risk factors for recurrence. If reaching a consensus was necessary, a third independent investigator was enrolled.

The 18 studies included in our analysis came from treatment programs from 13 countries and comprised of successfully treated patients with sample sizes ranging from 33 to 439. Four of these studies were conducted in hospital settings and 9 in community settings. The median age of patients ranged from 27.5 years to 47.5 years. Only one study applied re-initiation of treatment alone to the definition of recurrence. All other studies (n=12) applied culture positivity to the definition of recurrence in conjunction with smear positivity, re-initiation of treatment and radiographic deterioration. Recurrence rates ranged from 0% to 8.5% for the median duration of follow-up varied from 19.2 months to 98.1 months. Six studies provided factors independently associated with recurrence, including receiving only first-line drugs, XDR-TB, aggressive regimen <18 months, diabetes mellitus and outcome as treatment completion (vs. cured).

**Search algorithm**

PubMed

((("Cohort studies"[Mesh])) AND ((Recurrence[Mesh] OR recurrence*[tw] OR relapse*[tw] OR reinfection*[tw])) AND (("Tuberculosis, Multidrug-Resistant/drug therapy*"[Mesh] OR multidrug-resistant[tw]))

**Figure. Study selection process of literature review of MDR-TB recurrence**

189 articles from initial electronic database search

8 articles not meet inclusion criteria

4 had follow-up < 24 months

2 had patients with incomplete treatment

1 had no outcome (recurrence)

1 had no analysis on MDR-TB patients

1 article identified from bibliographic search

164 articles excluded based on title and abstract

25 articles retained for full text review

26 articles chosen for full text review

18 articles included in review

13 studies

**Table. Summary of studies included in the review of MDR-TB recurrence**

| Author, year (location) | Setting | Year of diagnosis or treatment of MDR-TB | Definition of recurrence (relapse or reinfection) | Patients eligible for recurrence observation / No. of MDR-TB with treatment success^a^ (%) | Mean or median age, years (range)^b^ | Mean or median duration of follow-up, months (range) | No. of recurrence | Recurrence rate^c^ (%) | Recurrence rate (per 1000 person-months) | Risk factors for recurrence |
| --- | --- | --- | --- | --- | --- | --- | --- | --- | --- | --- |
| Burgos et al, 2005 (San Francisco, USA) | Community | 1982–2000 | Relapse: positive culture | 32 / 33 (97) | 49.5 (22–78) | 24 (9–57) | 2 | 6.1 | NA | -- |
| Chiang et al, 2006 (Taipei, Taiwan) | Hospital (referral center) | 1992–1996  Follow-up until 2004 | Relapse: positive culture | 153 / 153 (100) | 47.3 (16–83) | 38.7 | 10 | 6.5 | 1.7 | Receiving only first-line drugs (cf. receiving second-line drugs including ofloxacin) |
| Karagöz et al, 2009 (Istanbul, Turkey) | Hospital (referral center) | 1995–2000 | Relapse: positive smear or culture | 89 / 102 (87) | 39 (16–65) | 19.2 (12–72) | 0 | 0^d^ | NA | -- |
| Leung et al, 2011 (Hong Kong) | Community | 1997–2006  Follow-up until 2009 | Relapse: positive culture (with DST), histological, radiographic (with improvement after re-treatment) | 187 / 187 (100) | NA | 68.4 | 8 | 4.3 | 0.6 | XDR-TB  (pre-XDR-TB or shorter treatment (≤15m): not significant) |
| Blondal et al, 2012 (Estonia) | Community | 2001–2003  Follow-up until 2010 | Recurrence: positive culture | 129 / 129 (100) | 42.9 | 98.1 (2.1–125.7)^e^ | 11 | 8.5 | NA | History of previous TB treatment |
| Anderson et al, 2013  (United Kingdom) | Community | 2004–2007  Follow-up until 2012 | Relapse: positive culture | 145 / 145 (100) | Mode: age group 15–44 | Minimum of 4 y | 1 | 0.7 | NA | -- |
| Franke et al, 2013 (Lima, Peru) | Community | 1999–2002 | Relapse:  a. Positive TB culture  b. Re-initiation of TB treatment  (only smear positivity not included) | 402 / 442 (91) | 27.5 (IQR 22.5–36.7)^f^ | 40.5 (21.2–53.4) | 26 | 6.5 | 1-year: 3.2  2-year: 0.5 | 1. Aggressive regimen^g^ <18 months  2. Diabetes mellitus |
| Aung et al, 2014  (Bangladesh) | Community | 2005–2011 | 1. Relapse: culture positive except reinfection  2. Reinfection: recurrent disease with a  genotypically different strain | 362 / 439 (82) | Mode: age group 31-42 | ≥82% of patients had 2-y follow-up, 20% had 5-y follow-up | 4 | 0.9 | NA | -- |
| Piubello et al, 2014  (Niger) | Community | 2008–2010 | Relapse: positive culture | 49 / 58 (85) | 31 (16–66) | ≥85% of patients had 2-y follow-up | 0 | 0 | NA | -- |
| Yoshiyama et al, 2014  (Tokyo, Japan) | Hospital | 1990–2011 | Relapse: positive culture | 168 / 168 (100) | 47 (18–99) | 52.8 | 3 | 1.8 | NA | -- |
| Gelmanova et al, 2015  (Tomsk, Russia) | Community | 2000– 2004 | Recurrence:  1. A sputum culture growing TB followed by another positive culture or death  2. MDR-TB treatment re-initiation regardless of culture results | 399 / 409 (98) | NA | 42.4 (IQR 20.5–59.5) | 27 | 6.8 | 1-year : 1.4  after 1-year : 1.8 | 1. Time and recurrence: not significant  2. Treatment completed cf. cured: not significant |
| Kuaban et al, 2015  (Cameroon) | Community | 2008–2011 | Relapse: positive culture | 100 / 134 (75) | 33.7 (17–68) | 87% of patients had 18-m follow-up, 75%, had 24-m follow-up | 0 | 0 | NA | -- |
| Kwak et al, 2015  (Seoul, South Korea) | Hospital | 2006–2010 | Relapse: re-initiation of treatment >6m | NA / 105 | 37 (27–56) | 38.7 (23–54)^h^ | 2 | 1.9 | 0.7 | Treatment completed (cf. cured)^h^ |

^a^ Including patients who were cured or completed treatment. ^b^ All except Frank et al.’s study referred to all patients who constituted the study population (including patients without treatment success). ^c^ Number of recurrence divided by number of MDR-TB patients with treatment success and eligible for recurrence observation. ^d^ 1.3% (1/78) in 2-year follow-up in 2001 study (Tahaoglu K et al., 2001). ^e^ Of 211 patients who constituted the study population; calculated from start of treatment with second-line drugs. ^f^ Of 402 patients with treatment success. ^g^ Aggressive regimen was one that contained at least 5 anti-tuberculosis agents which met suggesting efficacy according to the individual’s drug susceptibility testing and treatment history. ^h^ Data from 2011 study (Lee J et al, 2011).

IQR, interquartile range; MDR-TB, multidrug-resistant tuberculosis (resistance to at least isoniazid and rifampin); NA, not available; pre-XDR, MDR-TB plus resistance to any fluoroquinolone or any second-line injectable drug; TB, tuberculosis; XDR, extensively drug-resistant tuberculosis (MDR-TB plus resistance to any fluoroquinolone and any second-line injectable drug).
