# Supplementary material for: Recurrence after Successful Treatment of Multidrug-Resistant Tuberculosis in Taiwan
Source: PLoS One. 2017 Jan 26;12(1):e0170980. doi: 10.1371/journal.pone.0170980 (PMC5270331; doi:10.1371/journal.pone.0170980)
Supplement: S1 Table — (DOCX) [file pone.0170980.s002.docx]

**Table S1. Multivariate analyses of potential predictors of multidrug-resistant tuberculosis recurrence (n=295)**

|  | **MDT-TB patients**  **(n=295)** | | | **Sub-analysis of patients with second-line DST (n=215)** | |
| --- | --- | --- | --- | --- | --- |
|  | **Adjusted HR (95% CI)** | | **p value** | **Adjusted HR (95% CI)** | **p value** |
| Gender | |  |  |  |  |
| Male | | 3.6 (0.4–34) | 0.27 | 3.0 (0.3–30) | 0.35 |
| Female | | Reference |  | Reference |  |
| Age groups (years) | |  |  |  |  |
| >60 | | 0.6 (0.1–3.6) | 0.57 | 1.1 (0.1–8.5) | 0.95 |
| 35–60 | | 0.4 (0.1–1.6) | 0.19 | 0.7 (0.1–3.9) | 0.64 |
| <35 | | Reference |  | Reference |  |
| BMI (kg/m^2^) | |  |  |  |  |
| <18.5 | | 0.5 (0.03–9.7) | 0.64 | — | — |
| 18.5–25 | | 1.2 (0.1–10) | 0.89 |  |  |
| ≥25 | | Reference |  |  |  |
| Patient classification | |  |  |  |  |
| Treatment after failure of previous treatment | | 0.8 (0.1–6.2) | 0.85 | 1.7 (0.1–20)^b^ | 0.66 |
| Treatment after loss to follow-up | | 2.6 (0.2–32) | 0.45 |  |  |
| Relapse | | 2.6 (0.5–14) | 0.28 | 6.7 (0.7–66) | 0.11 |
| New | | Reference |  | Reference |  |
| Cavitation on initial CXR | |  |  |  |  |
| Yes | | 6.3 (1.2–34) | 0.03 | 10.2 (1.2–89) | 0.04 |
| No | | Reference |  | Reference |  |
| Sputum smear positivity at time of diagnosis | |  |  |  |  |
| Yes | | 0.8 (0.2–3.1) | 0.75 | — | — |
| No | | Reference |  |  |  |
| Treatment delay^a^ | |  |  |  |  |
| Yes | | 2.1 (0.5–8.1) | 0.30 | — | — |
| No | | Reference |  |  |  |
| Culture conversion before initiating SLD | |  |  |  |  |
| Yes | | 0.5 (0.1–2.5) | 0.40 | — | — |
| No | | Reference |  |  |  |
| Time from initiating SLD to culture conversion | |  |  |  |  |
| ≥2 months | | 0.8 (0.2–3.4) | 0.73 | 0.6 (0.1–3.2) | 0.54 |
| <2 months | | Reference |  | Reference |  |
| Second-line DST | |  |  |  |  |
| pre-XDR or XDR | | — | — | 7.3 (1.2–44) | 0.03 |
| MDR only | |  |  | Reference |  |

^a^ The lag between sputum collection of MDR-TB and start of second-line drugs >120 days.

^b^ Patient classifications as treatment after failure of previous treatment and as treatment after loss to follow-up were combined into one group.

BMI, body mass index; CI, confidence interval; CXR, chest radiography; DST, drug susceptibility test; HR, hazard ratio; MDR-TB, multidrug-resistant tuberculosis (resistance to at least isoniazid and rifampin); MDR only, MDR-TB but susceptible fluoroquinolones and second-line injectable drugs; pre-XDR, MDR-TB plus resistance to any fluoroquinolone or any second-line injectable drug; SLD, second-line drugs (include fluoroquinolones and second-line injectable drugs); XDR, extensively drug-resistant tuberculosis (MDR-TB plus resistance to any fluoroquinolone and any second-line injectable drug).
