# Supplementary material for: Recurrence after Successful Treatment of Multidrug-Resistant Tuberculosis in Taiwan
Source: PLoS One. 2017 Jan 26;12(1):e0170980. doi: 10.1371/journal.pone.0170980 (PMC5270331; doi:10.1371/journal.pone.0170980)
Supplement: S2 Table — (DOCX) [file pone.0170980.s003.docx]

**Table S2 Detailed information of drug susceptibility results in 10 patients with multidrug-resistant tuberculosis recurrence**

| Patient No. | Episode, isolate | Isoniazid | Rifampin | Ethambutol | Pyrazinamide | Streptomycin | SLI | FQ | Second-line DST |
| --- | --- | --- | --- | --- | --- | --- | --- | --- | --- |
| 1 | 1^st^, baseline | R | R | S | NA | S | S | NA | NA |
|  | 1^st^, final | R | R | S | NA | S | NA | NA | NA |
|  | 2^nd^, baseline | R | R | R | NA | R | S | R | Pre-XDR |
| 2 | 1^st^, baseline | R | R | S | NA | S | NA | R | Pre-XDR |
|  | 1^st^, final | R | R | R | NA | S | NA | NA | NA |
|  | 2^nd^, baseline | R | R | R | NA | S | NA | NA | NA |
| 3 | 1^st^, baseline | R | R | S | NA | S | NA | NA | NA |
|  | 1^st^, final | R | R | S | NA | S | S | S | MDR only |
|  | 2^nd^,baseline | R | R | S | S | R | S | S | MDR only |
| 4 | 1^st^, baseline | R | R | S | NA | R | S | S | MDR only |
|  | 2^nd^, baseline | R | R | R | S | R | S | S | MDR only |
| 5 | 1^st^, baseline | R | R | R | NA | S | S | R | Pre-XDR |
|  | 1^st^, final | R | R | R | NA | S | NA | NA | NA |
|  | 2^nd^, baseline | R | R | R | R | S | S | R | Pre-XDR |
| 6 | 1^st^, baseline | R | R | S | NA | S | R | S | Pre-XDR |
|  | 1^st^, final | R | R | S | NA | S | R | R | XDR |
|  | 2^nd^, baseline | R | R | R | NA | R | R | R | XDR |
| 7 | 1^st^, baseline | R | R | S | NA | R | S | S | MDR only |
|  | 1^st^, final | R | R | S | NA | R | S | S | MDR only |
|  | 2^nd^, baseline | R | R | NA | NA | NA | NA | NA | NA |
| 8 | 1^st^, baseline | R | R | S | NA | R | S | S | MDR only |
|  | 1^st^, final | R | R | R | NA | R | S | S | MDR only |
|  | 2^nd^, baseline | R | R | S | NA | R | S | S | MDR only |
| 9 | 1^st^, baseline | R | R | S | NA | R | S | NA | NA |
|  | 1^st^, final | R | R | S | NA | R | S | S | MDR only |
|  | 2^nd^, baseline | R | R | S | NA | R | NA | NA | NA |
| 10 | 1^st^, baseline | R | R | S | NA | S | S | S | MDR only |
|  | 1^st^, final | R | R | R | NA | R | S | R | Pre-XDR |
|  | 2^nd^, baseline | R | R | R | S | R | S | R | Pre-XDR |

DST, drug susceptibility test; FQ, fluoroquinolone; NA, not available; R, resistance; S, susceptible; SM, streptomycin, SLI, second-line injectable drugs; MDR-TB, multidrug-resistant tuberculosis (resistance to at least isoniazid and rifampin); MDR only, MDR-TB, but susceptible to fluoroquinolones and second-line injectable drugs; pre-XDR, MDR-TB plus resistance to any fluoroquinolone or any second-line injectable drug; XDR, extensively drug-resistant tuberculosis (MDR-TB plus resistance to any fluoroquinolone and any second-line injectable drug).
